# Surface Quality Improvement for Ultrasonic-Assisted Inner Diameter Sawing with Six-Axis Force Sensors

**DOI:** 10.3390/s23146444

**Published:** 2023-07-16

**Authors:** Jinghe Zhao, Lulu Wang, Bo Jiang, Yongchen Pei, Huiqi Lu

**Affiliations:** 1 School of Mechanical Engineering, Changchun Guanghua University, Changchun 130033, China; zhaojinghe@ghu.edu.cn (J.Z.); jiangbo@ghu.edu.cn (B.J.); 2 School of Mechanical and Aerospace Engineering, Jilin University, Changchun 130025, China; 3 FAW-Volkswagen Automobile Co., Ltd., Changchun 130011, China; 4 Institute of Biomedical Engineering, Department of Engineering Science, University of Oxford, Oxford OX3 7DQ, UK; yvonne.lu@eng.ox.ac.uk

**Keywords:** normal sawing force model, six-axis force sensor, inner diameter sawing, ultrasonic-assisted machining, alumina ceramics

## Abstract

Ultrasonic-assisted inner diameter machining is a slicing method for hard and brittle materials. During this process, the sawing force is the main factor affecting the workpiece surface quality and tool life. Therefore, based on indentation fracture mechanics, a theoretical model of the cutting force of an ultrasound-assisted inner diameter saw is established in this paper for surface quality improvement. The cutting experiment was carried out with alumina ceramics (99%) as an exemplar of hard and brittle material. A six-axis force sensor was used to measure the sawing force in the experiment. The correctness of the theoretical model was verified by comparing the theoretical modeling with the actual cutting force, and the influence of machining parameters on the normal sawing force was evaluated. The experimental results showed that the ultrasonic-assisted cutting force model based on the six-axis force sensor proposed in this paper was more accurate. Compared with the regular tetrahedral abrasive model, the mean value and variance of the proposed model’s force prediction error were reduced by 5.08% and 2.56%. Furthermore, by using the proposed model, the sawing processing parameters could be updated to improve the slice surface quality from a roughness Sa value of 1.534 µm to 1.129 µm. The proposed model provides guidance for the selection of process parameters and can improve processing efficiency and quality in subsequent real-world production.

## 1. Introduction

There are two main processing methods for hard and brittle material slicing: diameter sawing [1,2,3] and wire sawing [4,5,6,7]. The diameter sawing methods broadly include inner diameter sawing [8,9,10] and external diameter sawing [11,12]. Compared with wire sawing and external diameter sawing, inner diameter sawing technology has greater advantages in small batch production and wafer roundness control, and it is more suitable for the processing of ceramics with moderate or short lengths [13]. The inner diameter sawing technology realizes the slicing of ceramics using a thin and high-speed rotating diamond sawing blade with a center hole and a layer of emery plated on the inner edge of the center hole. However, existing research has shown that the surface roughness of slices obtained with traditional inner diameter sawing technology cannot meet the needs of market applications.

Ultrasonic vibration can considerably improve the surface quality of parts and is one of the most effective machining methods to reduce cutting force and cutting temperature. Yang Z. et al. found that ultrasonic vibration can effectively improve the surface quality of ZrO_2_ ceramic slices by comparing the surface quality of ultrasonic vibration-assisted grinding with that of ordinary grinding [14]. Wu H. Q. et al. found that ultrasonic vibration machining improved the surface quality and material removal rate for Ti-6Al-4V titanium alloy and discussed the mechanism by which ultrasonic vibration improved the machining performance [15]. Zhu X. X. performed ultrasound-assisted micro-hole drilling with a DD6 nickel-based superalloy. Compared with conventional drilling, ultrasound-assisted micro-hole drilling reduced the burr height, improved the processing accuracy, and increased the life of the bit [16]. Chen Y. ground sapphire and found that ultrasonic-assisted grinding improved the surface finish of the sapphire in the axial and tangential direction compared with ordinary grinding [17]. Muhammad Riaz applied ultrasonic vibration to a turning tool to study the influence of processing parameters on the surface roughness of titanium alloy and confirmed that the ultrasonic-assisted process could significantly reduce the cutting force and improve the sawing surface quality [18]. Sui H. et al. combined ultrasonic vibration-assisted machining and boring and found that the combined approach was effective in reducing the boring force and improving the accuracy of the boring. After processing the surface residual stress from the tensile state to the compression state, the surface anti-fatigue performance was greatly improved [19]. Zhao Y. et al. simulated and analyzed the dynamic cutting process of ultrasonic vibration-assisted drilling technology to improve rock-breaking efficiency for deep wells, and the results showed that the average cutting force was reduced by 50% [20]. The above findings indicate that ultrasonic-assisted machining can effectively reduce the sawing force in the cutting process and help to improve the surface quality of slices.

**Figure 1:**
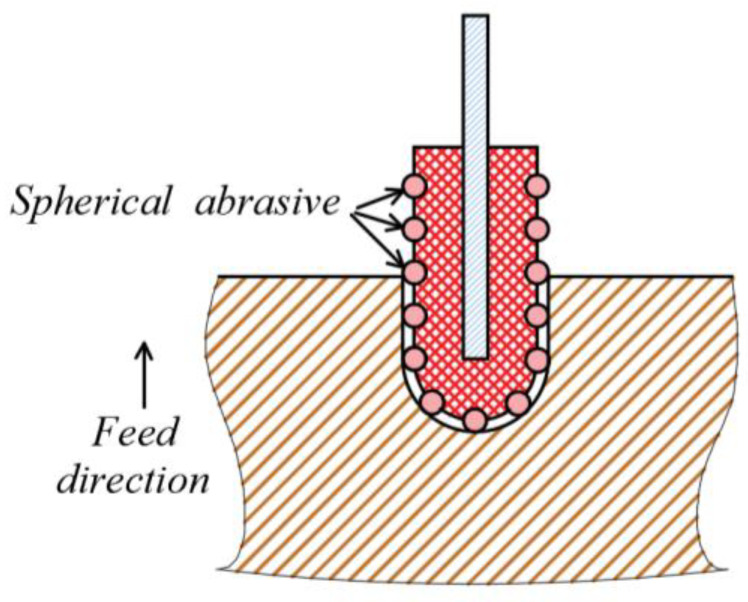
Ultrasonic-assisted inner diameter sawing schematic diagram.

As an advanced production technology, ultrasonic vibration-assisted machining has been widely used in the manufacture of hard and brittle material components. However, limited work has been undertaken in real-world settings to improve the mechanism by which ultrasonic vibration affects cutting force. It is important to model the sawing process and address the uncertainty and extreme scenarios in real-world settings while optimizing process parameters to further reduce cutting force. Ultrasonic vibration-assisted inner diameter machining has been studied, and the relationship between the sawing force and other parameters was described based on a regular tetrahedron model [21]. However, the shapes of the abrasive grains electroplated on the inner diameter sawing blade do not have a regular geometry, so the cutting force model error with regular tetrahedral abrasive grains is larger and needs further study. Differently from in previous work, the shape obtained by abrasive electroplating on the inner diameter saw blade was not a regular hexahedron, as shown in Figure 1.

Ceramics are typically hard and brittle materials with the advantages of being lightweight and having high temperature resistance, oxidation resistance, and corrosion resistance. Therefore, ceramic materials are widely used in aerospace components, such as insulation tiles, turbine blades, and inner combustion engine parts [22,23]. Ceramic matrix composite materials are a key issue restricting the development of aerospace vehicle manufacturing technology in the future. Therefore, how to efficiently obtain a ceramic matrix with high quality and high surface accuracy is particularly important.

**Figure 2:**
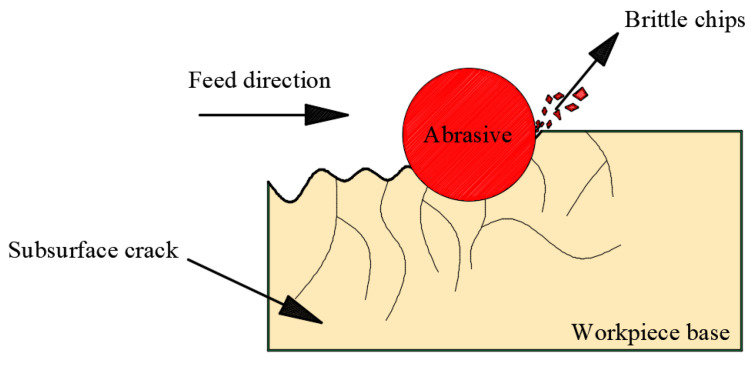
Schematic diagram of ceramic processing.

The objective of this study was to develop an intelligent sensor-based ultrasonic-assisted inner diameter saw cutting force system. With the development of sensors, temperature sensors [24], image sensors [25], and force sensors have appeared [26] more and more frequently, allowing performance and quality improvements in industrial applications. A six-axis force sensor was integrated for force measuring. In Section 2, the normal sawing force model was built for ultrasonic vibration-assisted precision machining using inner diameter sawing with ceramics. The material removal process is shown in Figure 2. The theoretical relationship between the normal sawing force and other process parameters was obtained, and how key sawing parameters affect the maximum normal sawing force was identified. The differences between the simulation results from the theoretical model and the experiment test results were measured and analyzed. Finally, the association between the normal sawing force and the surface quality of alumina ceramic slices was analyzed and evaluated.

In this study, a new ultrasonic-assisted sawing force model for an internal diameter sawing machine was established by using a six-axis force sensor to measure the sawing force in the experiment and associating the cutting force with the slice surface quality according to the experimental data obtained.

## 2. Development of the Model of the Inner Diameter Sawing System

There is no plastic deformation for an ideal hard and brittle material. Therefore, the workpiece is modified through the expansion and crossing of the diamond abrasive grain against the inner diameter of the sawing blade. To establish the normal sawing force, the model assumptions were set as follows.

The properties and dimensions of the alumina ceramic materials involved in the experiment were consistent. Brittle fracture was the main removal method for hard and brittle materials. The diamond abrasive grain on the inner diameter edge of the inner diameter sawing blade was considered an ideal rigid sphere. All these properties affected the cutting.

### 2.1. Sawing Depth Modeling

In this study, the Hertz equation was used to estimate the normal sawing force and the relationship between the cutting depth and the magnitude of the normal sawing force, as shown in Equation (1). The relationship between the maximum sawing depth and the normal sawing force was established as follows [27]:

(1) $$\gamma ={\left[\frac{9}{16}\frac{{\left(F_{hn}\right)}^{2}}{r_{m}}{\left(\frac{1-\nu_{t}^{2}}{E_{t}}+\frac{1-\nu_{j}^{2}}{E_{j}}\right)}^{2}\right]}^{1/3}$$

where *γ* is the sawing depth (in mm); *F_hn_* is the maximum impact force between a single diamond abrasive grain and the workpiece (in N); *r_m_* is the radius of rigid spherical abrasive particles (in mm); *ν_j_* is the Poisson ratio for alumina ceramics; *ν_t_* is the Poisson ratio for diamond abrasive grains; *E_j_* is the elastic modulus of the alumina ceramics (MPa); and *E_t_* is the elastic modulus for the diamond abrasive grains (in MPa). As the elastic modulus of diamond abrasive grains was much higher than that of alumina ceramics (*E_t_* >> *E_j_*) and $1-v_{j}^{2}\leq 1$, Formula (1) was simplified to:

(2) $$\gamma ={\left[\frac{9}{16}\frac{{\left(F_{hn}\right)}^{2}}{r_{m}}{\left(\frac{1-\nu_{j}^{2}}{E_{j}}\right)}^{2}\right]}^{1/3}$$

### 2.2. Normal Sawing Force

**Figure 3:**
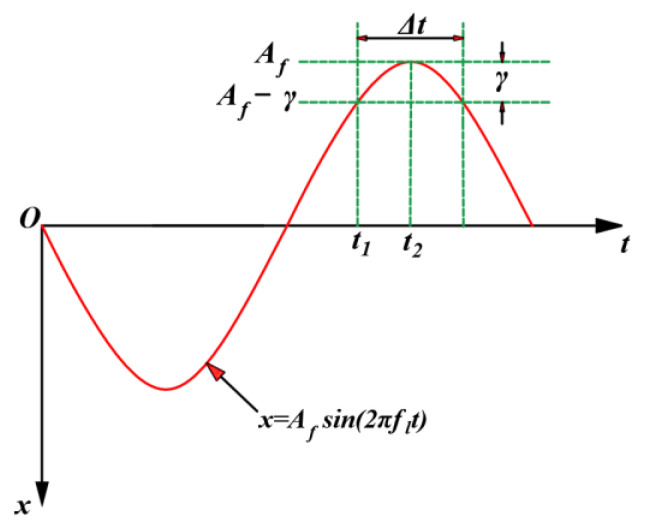
Effective sawing time for ultrasonic vibration-assisted inner diameter sawing.

In the process of ultrasonic vibration-assisted inner diameter sawing, the existence of ultrasonic vibration makes the blade and the workpiece intermittently enter into contact with each other; therefore, the cutting force is reduced during the cutting process. The normal sawing force increases and potentially reaches its maximum with the increase in sawing depth. With the deepening of the inner diameter sawing blade, the number of active abrasive particles involved in cutting constantly changes. The normal sawing force of a single abrasive particle was first estimated using Formula (3).

(3) $$F_{hm}=\Delta tf_{l}F_{hn}$$

where *F_hm_* is the normal sawing force of a single abrasive particle, which is the force (in N) in the *X*-axis direction in Figure 3; Δ*t* is the effective sawing time (in seconds); and *f_l_* is the frequency of ultrasonic vibration in the normal direction (in Hz). The movement of the diamond abrasive grain electroplated on the inner diameter sawing blade is mainly determined by the amplitude of ultrasonic vibration *A_f_* (in mm) and the frequency of the ultrasonic vibration *f_l_*, and the trajectory of the ultrasonic vibration can be described as a sinusoidal wave. The average position of the abrasive particles relative to themselves can be expressed by the following formula:

(4) $$y=A_{f}\sin(2\pi f_{l}t)$$

According to Figure 3, the effective cutting time Δ*t* represents the position of the diamond abrasive grain from x = *A_f_ − γ* to x = *A_f_*. The specific expression is as follows:

(5) $$\Delta t=2(t_{2}-t_{1})=\frac{1}{\pi f_{l}}\left[\frac{\pi }{2}-\arcsin(1-\frac{\gamma }{A_{f}})\right]$$

### 2.3. Active Abrasive Particle Modeling

According to the definition of abrasive particle concentration, the number of abrasive particles on the inner diameter sawing blade can be theoretically estimated. The concentration of abrasive particles was calculated based on their weight. If the abrasive concentration is 100, then every cubic millimeter of the volume contains 0.88 × 10^−3^ abrasive grains. The number of active abrasive particles on the cutting edge of the inner diameter blade was calculated according to the diamond abrasive particle size, abrasive particle concentration, and blade size. In our model, the abrasive particles were simplified as rigid spheres, so the volume of a single abrasive particle was $\frac{4}{3}\pi r_{m}^{3}$. The abrasive particles were considered evenly distributed on the inner diameter sawing blade based on the previous assumption, and the number of abrasive particles on the inner edge of the inner diameter sawing blade could be determined with the following formula:

(6) $$N_{h}={\left[\frac{0.88\times 10^{-3}}{(4/3)\pi r_{m}{}^{3}\rho }\frac{C_{a}}{100}\right]}^{2/3}A_{p}$$

In Formula (6), *C_a_* is the concentration of abrasive particles on the inner edge of the inner diameter blade, and *ρ* is the density of the diamond abrasive particles (g/mm^3^). The density of the spherical diamond abrasive particles *ρ* = 3.52 × 10^−3^ g/mm^3^. *A_p_* is the area (in mm^2^) of the inner diameter sawing blade insert involved in the cutting.

**Figure 4:**
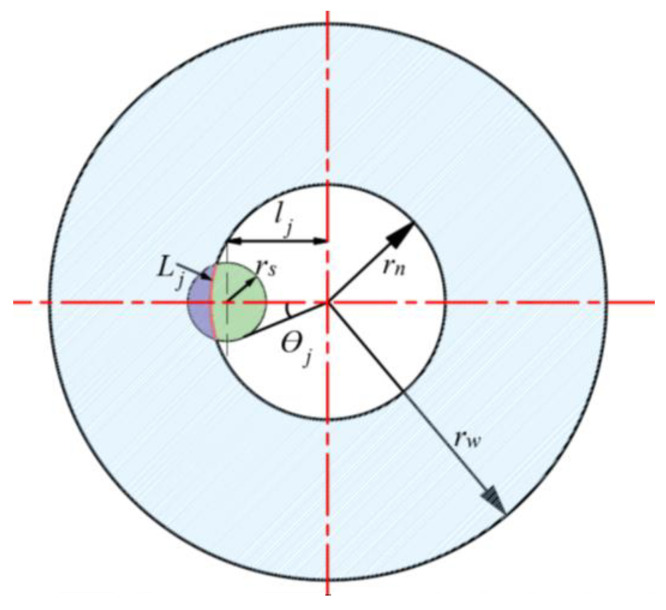
The central angle of a single abrasive particle during the sawing process with ultrasonic-assisted inner diameter sawing.

With the continuous increase in the sawing depth in the process of ultrasonic vibration-assisted inner diameter machining, the area of the inner diameter sawing blade participating in the sawing changed. Therefore, a theoretical calculation of the area of the inner diameter sawing blade participating in the cutting was carried out. Firstly, we derived the central angle during the sawing process using the law of cosines [2]:

(7) $$\theta_{j}=\arccos\left[1-\frac{2\delta (1-\delta )\eta^{2}}{1+(2\delta -1)\eta }\right]$$

where *δ* is the degree of cutting, $0<\delta \left(t\right)<1$, and *η* is a dimensionless parameter determined by the radius of the workpiece and the diameter of the blade that is numerically equal to the ratio of the two, 0<η<1, as shown in Figure 4. It was further deduced that the sawing area of the inner diameter sawing blade was as follows:

(8) $$A_{p}=\pi r_{n}\theta_{j}h_{n}$$

where *h_n_* is the thickness of the inner diameter sawing blade (in mm) and *r_n_* is the inner diameter of the inner diameter sawing blade (in mm). Inserting Formulas (7) and (8) into Formula (6), the number of active abrasive particles on the inner edge of the inner diameter sawing blade can be obtained as follows:

(9) $$N_{h}=\pi r_{n}h_{n}{\left[\frac{0.88\times 10^{-3}}{(4/3)\pi r_{m}^{3}\rho }\frac{C_{a}}{100}\right]}^{2/3}\arccos\left[1-\frac{2\delta (1-\delta )\eta^{2}}{1+(2\delta -1)\eta }\right]$$

### 2.4. Fracture Removal Volume

**Figure 5:**
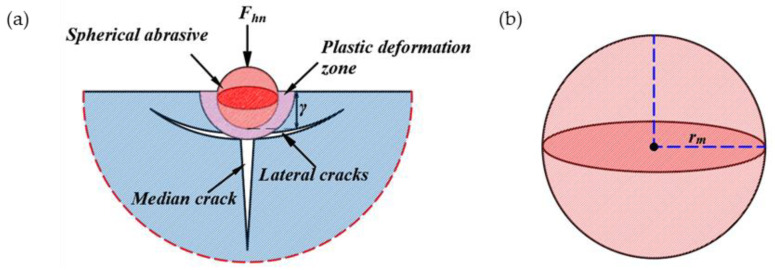
Ultrasonic-assisted inner diameter sawing indentation and spherical abrasive particles. (**a**) Model of indentation produced by spherical abrasive particles; (**b**) model of spherical abrasive particles.

To understand this sawing process, it is necessary to analyze the interaction between the abrasive particles and the workpiece. Figure 5a presents the brittle fracture material removal mode. The permanent plastic deformation zone is first formed by the load at the contact area between the spherical abrasive particles and the workpiece. As the load increases up to a certain critical value, the permanent plastic deformation zone gradually increases. Then, two transverse cracks are generated. When the spherical abrasive particles move to the next position, the cracks continue to expand, which eventually causes the material to fall from the workpiece. In this case, material removal is caused by plastic deformation and transverse cracks and mainly involves a brittle fracture mechanism.

**Figure 6:**
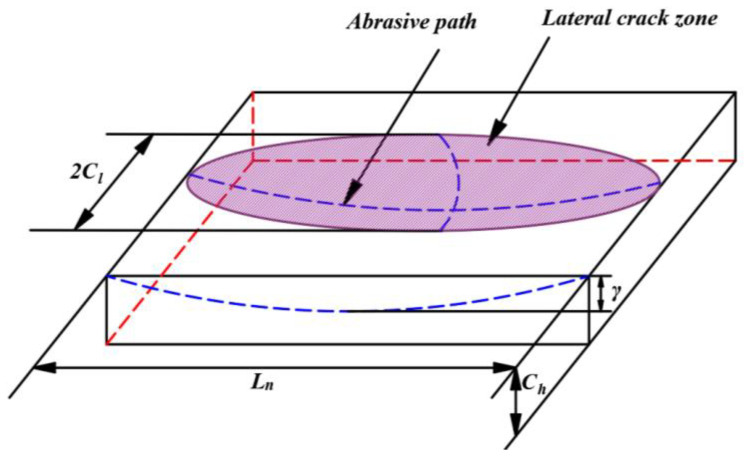
A fracture zone produced by an abrasive particle in ultrasonic-assisted inner diameter sawing.

The abrasive particles on the section of the inner diameter sawing blade move along a sine wave. Within the effective sawing time Δ*t*, the indentation of the ultrasonic vibration abrasive particles increases from 0 to *γ* and then decreases to 0. With the rotation of the tool, the abrasive particles slide a distance *L_n_* on the surface of the workpiece, as shown in Figure 6. The length and width of the fracture zone of the lateral crack also increase from 0 to the maximum and then decrease to 0. Until transverse cracks are formed, the material will be removed. The fracture area of a single abrasive grain is shown in Figure 6. The removed area can be simplified to a semi-elliptical volume with lengths *C_l_*, *C_h_*, and *L_n_*/2 with three semi-axes. Therefore, the removal volume *V_q_* for a single abrasive particle can be calculated with the following formula:

(10) $$V_{q}=\frac{1}{3}\pi C_{l}C_{h}L_{n}$$

where *C_l_* is the length of the lateral crack (in mm), *C_h_* is the depth of the lateral crack (in mm), and *L_n_* is the effective cutting distance (in mm) of the abrasive particles in the effective cutting time. The effective cutting distance *L_n_* (in mm) can be calculated with the following formula:

(11) $$L_{n}=\frac{r_{n}W}{30f_{l}}\left[\frac{\pi }{2}-\arcsin(1-\frac{\gamma }{A_{f}})\right]$$

where *W* is the spindle rotation speed; that is, the rotation speed of the inner blade (in r/min). The length of the lateral crack *C_l_* and the depth of the lateral crack *C_h_* can be determined using Formulas (12) and (13) [28]:

(12) $$C_{l}={\left(\frac{F_{hn}}{k_{c}}\right)}^{3/4}$$

(13) $$C_{h}={\left(\frac{F_{hn}}{H_{v}}\right)}^{1/2}$$

where *k_c_* is the plane strain fracture toughness coefficient (in MPa m^1/2^), and *H_v_* is the Vickers hardness of the material (in HV). Inserting Formulas (11)–(13) into Formula (10), the theoretical material removal volume *V_q_* can be obtained as follows:

(14) $$V_{q}=\frac{\pi }{90f_{l}}\frac{r_{n}WF_{hn}^{5/4}}{k_{c}^{3/4}H_{v}^{1/2}}\left[\frac{\pi }{2}-\arcsin(1-\frac{\gamma }{A_{f}})\right]$$

Once the indentation volume for each cycle of a single abrasive particle has been obtained, the relationship between the fracture volume and the indentation volume is known and the material removal rate can be predicted. Due to the complexity of this relationship and the diversity of factors affecting it, this relationship is not discussed in the literature. In this paper, these influencing factors are integrated into a single parameter, and the expression for the actual removal volume is as follows:

(15) $$V_{z}=K_{v}V_{q}$$

The proportional constant may be a function of material properties, process parameters, and the probability of causing a fracture. In order to further estimate the material removal rate, for a given material, *K_v_* must be kept constant within a wide range of process parameters; that is, *K_v_* is regarded as a constant in this paper.

The material removal rate can be theoretically calculated from the sum material removal rate *MRR* of all abrasive particles on the end face of the tool:

(16) $$MRR=N_{h}f_{l}V_{z}$$

In addition, according to the definition of the material removal rate, the material removal rate in ultrasonic vibration-assisted inner diameter sawing can also be expressed in terms of contact length, inner diameter sawing blade thickness, and feed speed. The specific expression is as follows:

(17) $$MRR=2f_{j}r_{n}\theta_{j}h_{n}$$

where *f_j_* is the feed rate of the machine tool (in mm/s). Combining Formulas (16) and (17), another expression of *F_hn_* (in N) can be obtained:

(18) $$F_{hn}=C_{2}\frac{f_{l}^{4/5}r_{m}^{8/5}\rho^{8/15}k_{c}^{3/5}H_{v}^{2/5}}{K_{v}^{4/5}r_{n}^{4/5}W^{4/5}C_{a}^{8/15}}{\left[\frac{\pi }{2}-\arcsin(1-\frac{\gamma }{A_{f}})\right]}^{-\frac{4}{5}}$$

where *C*_2_ is 1.0885 × 10^4^. Considering the influence of all active abrasive particles, the normal sawing force *F*_h_ (in N) can be calculated with the following formula:

(19) $$F_{h}=F_{hm}N_{h}$$

By incorporating Formulas (2), (3), and (9) into Formula (19):

(20) $$F_{h}=C_{3}\frac{r_{n}h_{n}C_{a}^{2/3}\gamma^{3/2}E_{j}}{r_{m}^{3/2}\rho^{2/3}(1-v_{j}^{2})}\left[\frac{\pi }{2}-\arcsin(1-\frac{\gamma }{A_{f}})\right]\arccos\left[1-\frac{2\delta (1-\delta )\eta^{2}}{1+(2\delta -1)\eta }\right]$$

where *C*_3_ = 2.187 × 10^−4^, another expression for *F_h_* can be written out:

(21) $$\begin{array}{l} F_{h}=C_{4}\frac{r_{n}^{1/5}h_{n}C_{a}^{2/15}f_{j}^{4/5}k_{c}^{3/5}H_{v}^{2/5}}{K_{v}^{4/5}r_{m}^{2/5}\rho^{2/15}W^{4/5}}\times \\ \;\;{\left[\frac{\pi }{2}-\arcsin(1-\frac{\gamma }{A_{f}})\right]}^{\frac{1}{5}}\arccos\left[1-\frac{2\delta (1-\delta )\eta^{2}}{1+(2\delta -1)\eta }\right] \end{array}$$

where *C*_4_ = 1.7854. Through Formula (21), the sawing depth *γ* can be determined and, by inserting the result of the sawing depth *γ* into Formula (20), the theoretically calculated normal sawing force can be obtained.

## 3. Sawing Experiment with Alumina Ceramics

### 3.1. Experiment Setup

**Figure 7:**
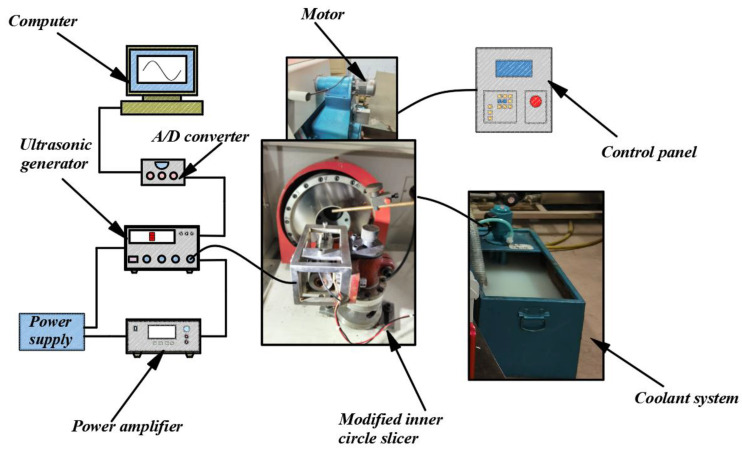
Ultrasonic vibration-assisted inner diameter sawing device.

The experiment in this study was based on the 5060 automatic inner diameter slicer, as shown in Figure 7. Based on this inner diameter slicer, a series of transformations were carried out. First, the ultrasonic vibrator was designed, including the transducer and horn, which played the main role, and finite element analysis was performed to determine the vibration node and resonance frequency. Second, the ultrasonic transmitter and power amplifier were connected to complete the overlap of the entire ultrasonic vibrator. Then, in order to facilitate the application of ultrasonic vibration, a fixing device for the workpiece was designed. By binding the workpiece to a wooden pad and then binding the wooden pad to the clamping device, this method makes material replacement more effective and convenient during the experimental process. An ADVANTECH PCIE-1816 acquisition card with a data sampling frequency of 5000 S/s was used to filter the signal measured by the six-axis force sensor through the median filtering method. Next, data acquisition cards and six-axis force sensors were installed to collect the cutting force data and measure the sawing movements during the experiment. The collected cutting force data were then compared with the simulated results from the theoretical model developed in Section 2. Details of the design of the ultrasonic vibrator and the clamping method of the workpiece can be found in our previous paper [29].

### 3.2. Experimental Design

If the value of fracture volume factor is a parameter independent of other factors, then, theoretically, only one set of experimental data can be used to calculate the specific value of *K_v_*. However, a series of experiments need to be performed to verify that *K_v_* is an independent parameter. Four variables were proposed in the experimental design: spindle speed, feed speed, abrasive granularity, and ultrasonic amplitude, as shown in Table 1. In this experiment, SPSS software was used to design the orthogonal experiment, and a total of 25 groups of experiments were completed.

## 4. Model Validation and Discussion

### 4.1. Calculation of the Fracture Toughness Coefficient

The unknown quantity of the fracture toughness coefficient *K_v_* was calculated with the following formula:

(22) $$K_{v}=\frac{V_{z}}{V_{q}}$$

The theoretical removal volume *V_q_* can be directly calculated with Formula (17), and the actual removal volume *V_z_* can be further calculated by calculating the material removal rate. Then, we calculated the actual removal volume *V_z_* with the following formula:

(23) $$V_{z}=\frac{MRR}{N_{h}f_{l}}$$

**Figure 8:**
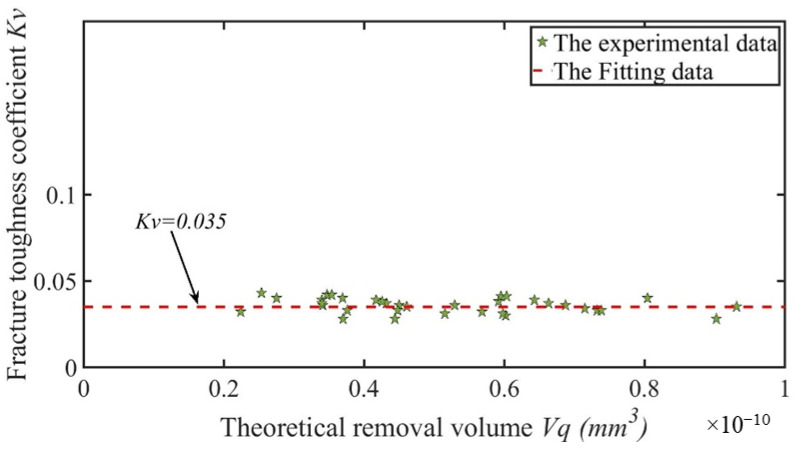
Fitting calculation for *K_v_*.

Then, the fracture toughness coefficient *K_v_* can be calculated with Formula (22) using the actual removal volume *V_z_* and theoretical removal volume *V_q_*. A more accurate value for the fracture toughness coefficient *K_v_* can be calculated through multiple sets of experiments. The data obtained were fitted, and the calculation results showed that *K_v_* was equal to 0.035, as shown in Figure 8. The experimental results showed that the value of *K_v_* was independent of other parameters.

### 4.2. Modeling of Normal Sawing Force

Substituting the calculated fracture toughness coefficient into Formula (24), the final expression of the normal sawing force was obtained as follows:

(24) $$\begin{array}{l} F_{h}=C_{5}\frac{r_{n}^{1/5}h_{n}C_{a}^{2/15}f_{j}^{4/5}k_{c}^{3/5}H_{v}^{2/5}}{r_{m}^{2/5}\rho^{2/15}W^{4/5}}\times \\ \;\;{\left[\frac{\pi }{2}-\arcsin(1-\frac{\gamma }{A_{f}})\right]}^{\frac{1}{5}}\arccos\left[1-\frac{2\delta (1-\delta )\eta^{2}}{1+(2\delta -1)\eta }\right] \end{array}$$

where *C*_5_ = 26.12. By comparing the theoretical normal sawing force with the actual normal sawing force measured by the six-axis force sensor in the actual cutting experiment, the error rate of this model was obtained.

**Figure 9:**
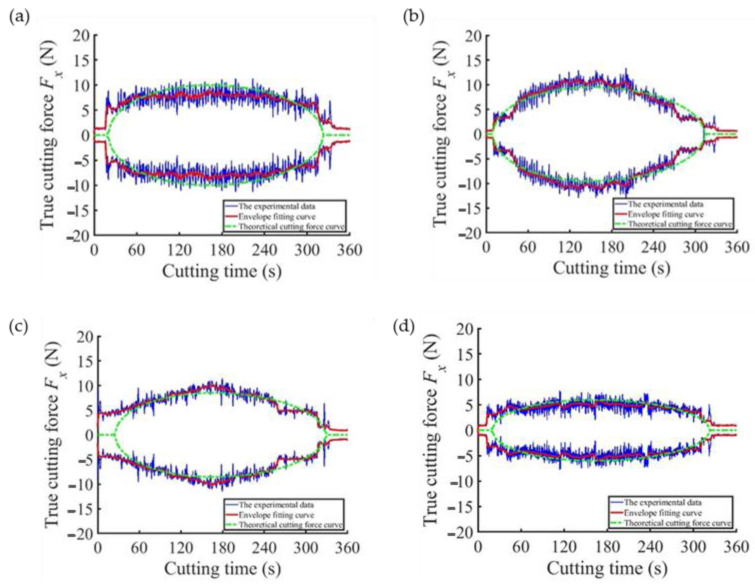

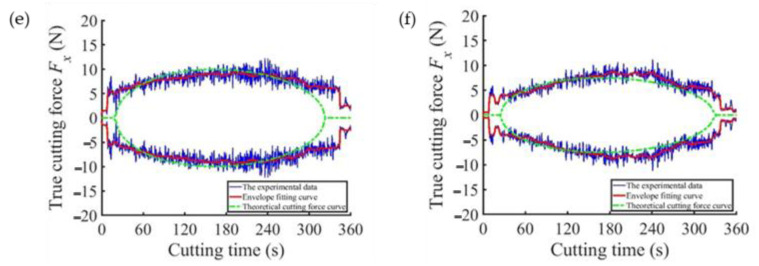
Comparison of experimental data and fitting curves. (**a**) *f_j_* = 0.033 mm/s, *r_m_* = 0.097 mm, *A_f_* = 3.2 μm, *r_n_* = 45 mm, *h_n_* = 0.5 mm, *W* = 3000 r/min; (**b**) *f_j_* = 0.0633 mm/s, *r_m_* = 0.097 mm, *A_f_* = 3.2 μm, *r_n_* = 45 mm, *h_n_* = 0.5, *W* = 3200 r/min; (**c**) *f_j_* = 0.033 mm/s, *r_n_* = 0.085 mm, *A_f_* = 6 μm, *r_n_* = 41.5 mm, *h_n_* = 0.4 mm, *W* = 3200 r/min; (**d**) *f_j_* = 0.033 mm/s, *r_n_* = 0.097 mm, *A_f_* = 3.2 μm, *r_n_* = 45 mm, *h_n_* = 0.5, *W* = 2400 r/min; (**e**) *f_j_* = 0.033 mm/s, *r_n_* = 0.097 mm, *A_f_* = 6 μm, *r_n_* = 45 mm, *h_n_* = 0.5 mm, *W* = 3000 r/min; (**f**) *f_j_* = 0.033 mm/s, *r_n_* = 0.065 mm, *A_f_* = 6 μm, *r_n_* = 41.5 mm, *h_n_* = 0.3, *W* = 3000 r/min.

**Figure 10:**
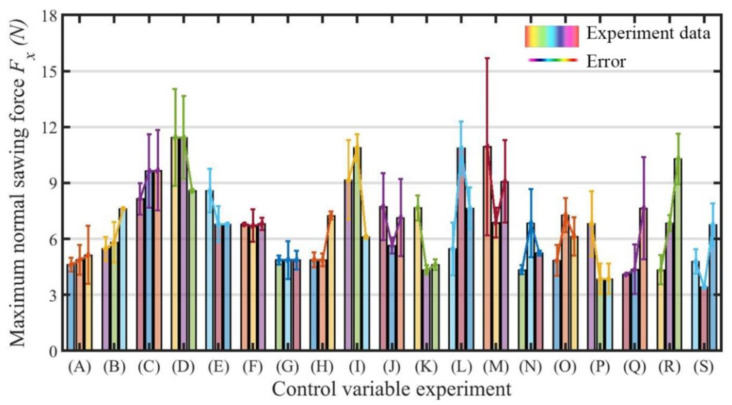
Error analysis of the normal sawing force model with rigid spherical abrasive particles.

The differences between all the experimental and simulation results are compared in Figure 9. The blue line represents the cutting force data measured in the actual machining process, the red line was obtained by enveloping and filtering the cutting force data, and the green dotted line is the cutting force curve obtained after bringing the corresponding machining parameters into the cutting force model. It can be seen from Figure 9 that the data measured in the experiment were consistent with the normal sawing force model calculated in theory. Then, part error analysis was performed with the obtained data, as shown in Figure 10. In 46 groups of sawing experiments, the average error reached 12.51%, and the maximum error was controlled at approximately 20%. As a result, there was little difference between the theoretical normal sawing force model and the actual measured normal sawing force, meaning that the actual normal sawing force could be predicted after using the theoretical model. Compared with the average error in the regular tetrahedral abrasive particle model, which is 16% [21], the model in this paper reduces the error by 3.49%.

**Figure 11:**
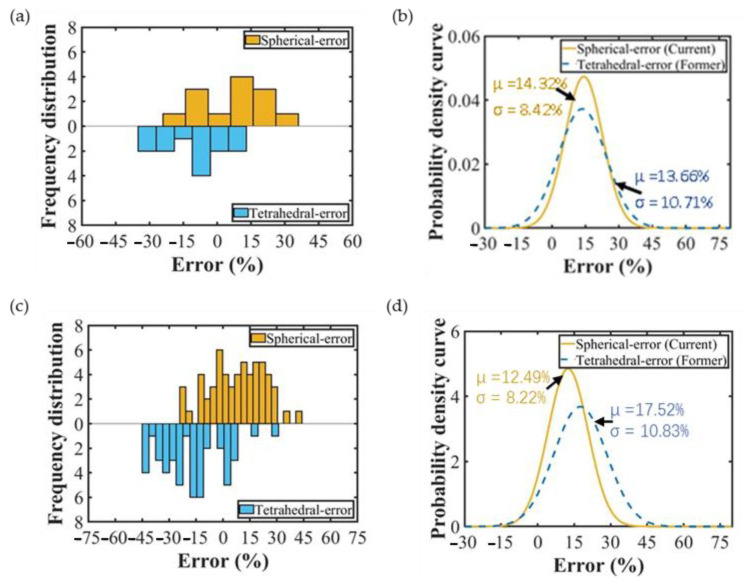
Error analysis of the maximum normal sawing force. (**a**) Error analysis of the monocrystalline silicon sawing experiment [21], (**b**) error statistical analysis of monocrystalline silicon [21], (**c**) error analysis of the alumina ceramic sawing experiment, (**d**) statistical error analysis of alumina ceramics.

The parameters of single-crystal silicon were brought into the normal sawing force model with spherical abrasive particles in order to compare it with our previous work and verify that the spherical abrasive normal sawing force model is more accurate than the regular tetrahedral abrasive normal sawing force model. To undertake a comparative analysis with the data from the known literature [21], the errors for 20 groups of data were calculated. The statistical error results for the two models can be seen in Figure 11a, and the statistical results were fitted to obtain Figure 11b. However, the blade vibration made the advantages of the model in this paper not obvious. There was little difference between the mean values of the two models, but the model proposed in this paper had a more concentrated error distribution than previous models, as shown in Figure 11b. Similarly, the sawing experiment data for 58 groups of alumina ceramics were brought into the two models. Figure 11c shows the error statistics for the two groups of different models, and it is obvious from Figure 11d that the mean value and variance of the proposed model’s perdition error were reduced by 5.08% and 2.56%, respectively, compared to the regular tetrahedral abrasive normal force model. It was verified that the model proposed in this paper is better than the regular tetrahedral abrasive particle model.

We found that the influence of some parameters on the maximum cutting force was larger based on multiple sets of experimental data processing and analysis and regular statistics, such as the feed rate and spindle speed, while the influence of other parameters on the maximum cutting force was small, such as the ultrasonic amplitude and particle size, because the thickness of the cutting edge depends on the size of the abrasive grain. Therefore, the influence of the cutting-edge thickness on the maximum sawing force was the same as that of the abrasive particle size.

**Figure 12:**
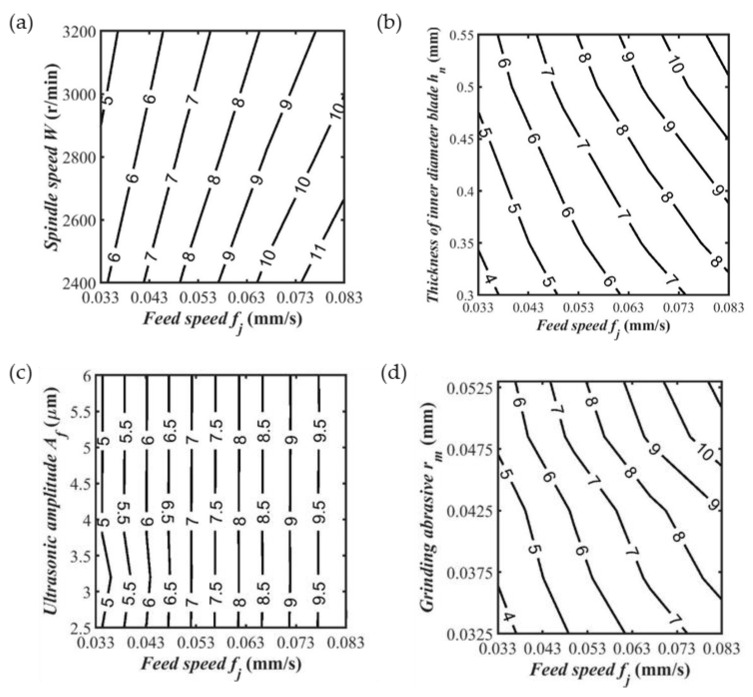
The influence of cutting parameters on the maximum normal sawing force. (**a**) *r_m_* = 0.097 mm, *A_f_* = 3.2 μm. *r_n_* = 45 mm, *h_n_* = 0.5 mm; (**b**) *A_f_* = 3.2 μm, *W* = 3000 r/min, *r_n_* = 41.5 mm; (**c**) *r_m_* = 0.097 mm, *r_n_* = 45 mm, *h_n_* = 0.5 mm, *W* = 3000 r/min; (**d**) *A_f_* = 3.2 μm, *W* = 3000 r/min, *r_n_* = 41.5 mm.

The influence of cutting parameters on the maximum normal sawing force was obtained by analyzing the experimental data. As shown in Figure 12a, the maximum normal sawing force decreased with increasing spindle speed. As shown in Figure 12b, the maximum normal sawing force increased with the increasing thickness of the inner diameter blade. As shown in Figure 12c, the maximum normal sawing force was almost unaffected by the ultrasonic amplitude. As shown in Figure 12d, the maximum normal sawing force increased with increasing abrasive particle size. Within the considered range of the research parameters, the feed speed and spindle speed had a great influence on the maximum normal sawing force; the grain size and the thickness of the inner diameter blade had little influence; and the ultrasonic amplitude had little effect on the maximum normal sawing force. In production activities, when the feed speed is lower, the spindle speed is higher, the inner diameter blade thickness is thinner, the grinding abrasive particle size is smaller, the maximum normal sawing force is smaller, and the quality of the slices obtained is better.

### 4.3. Normal Sawing Force and Surface Quality

**Figure 13:**
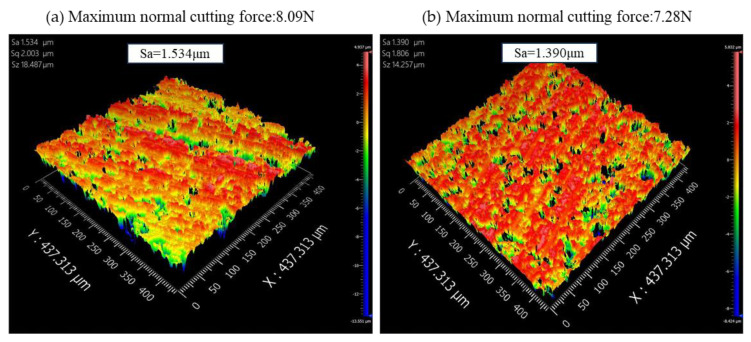

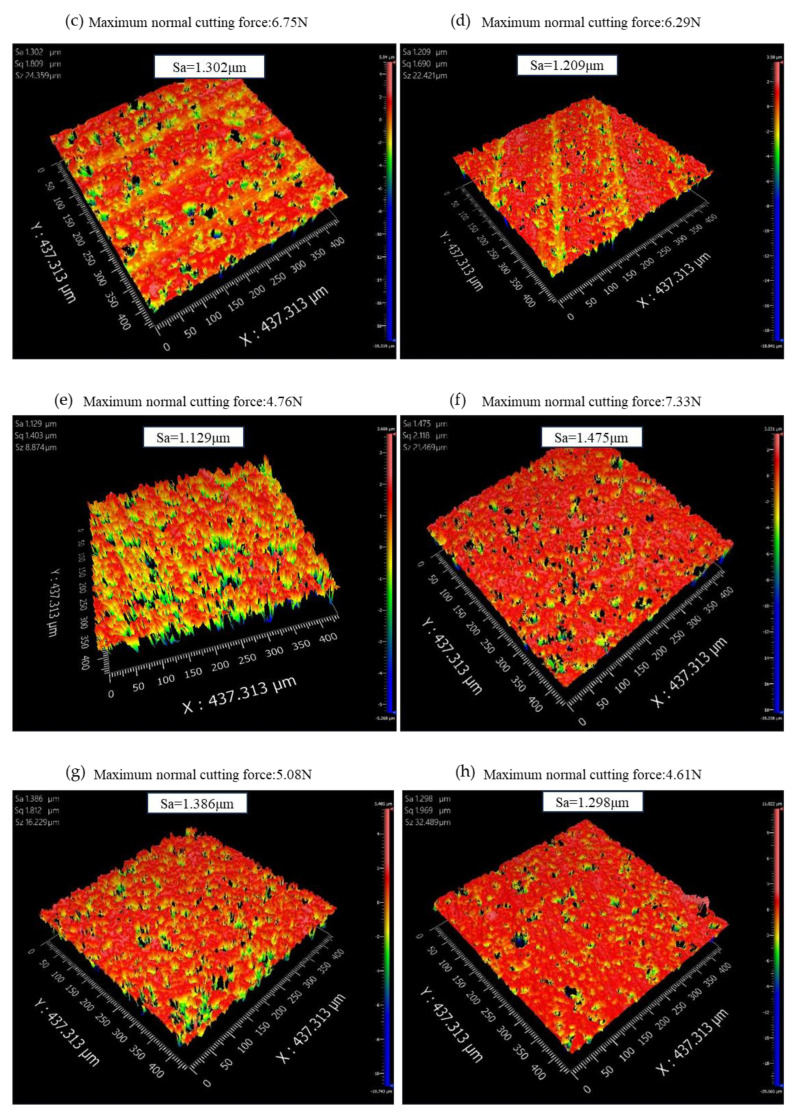

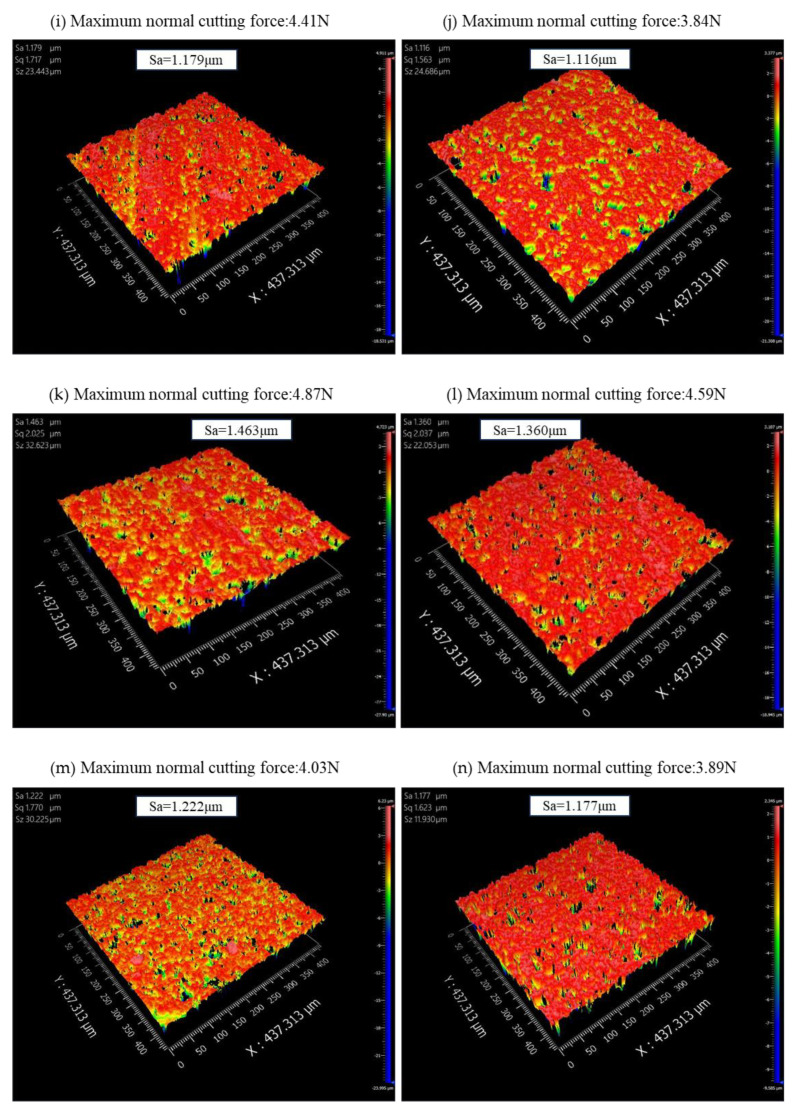

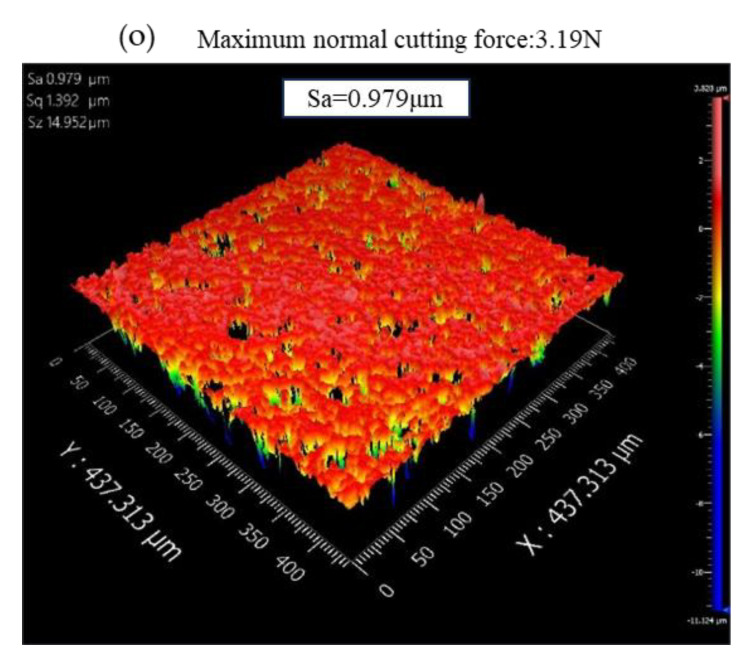
Relationship between slice surface quality and normal maximum sawing force. (**a**) *v* = 0.083 mm/s, *W* = 2400 r/min, *r_m_* = 0.097 mm, *r_n_* = 45 mm, *h_n_* = 0.5 mm; (**b**) *v* = 0.083 mm/s, *W* = 2600 r/min, *r_m_* = 0.097 mm, *r_n_* = 45 mm, *h_n_* = 0.5 mm; (**c**) *v* = 0.083 mm/s, *W* = 2800 r/min, *r_m_* = 0.097 mm, *r_n_* = 45 mm, *h_n_* = 0.5 mm; (**d**) *v* = 0.083 mm/s, *W* = 3000 r/min, *r_m_* = 0.097 mm, *r_n_* = 45 mm, *h_n_* = 0.5 mm; (**e**) *v* = 0.083 mm/s, *W* = 3200 r/min, *r_m_* = 0.097 mm, *r_n_* = 45 mm, *h_n_* = 0.5 mm; (**f**) *v* = 0.067 mm/s, *W* = 2400 r/min, *r_m_* = 0.097 mm, *r_n_* = 45 mm, *h_n_* = 0.5 mm; (**g**) *v* = 0.067 mm/s, *W* = 2600 r/min, *r_m_* = 0.097 mm, *r_n_* = 45 mm, *h_n_* = 0.5 mm; (**h**) *v* = 0.067 mm/s, *W* = 2800 r/min, *r_m_* = 0.097 mm, *r_n_* = 45 mm, *h_n_* = 0.5 mm; (**i**) *v* = 0.067 mm/s, *W* = 3000 r/min, *r_m_* = 0.097 mm, *r_n_* = 45 mm, *h_n_* = 0.5 mm; (**j**) *v* = 0.067 mm/s, *W* = 3200 r/min, *r_m_* = 0.097 mm, *r_n_* = 45 mm, *h_n_* = 0.5 mm; (**k**) *v* = 0.050 mm/s, *W* = 2400 r/min, *r_m_* = 0.097 mm, *r_n_* = 45 mm, *h_n_* = 0.5 mm; (**l**) *v* = 0.050 mm/s, *W* = 2600 r/min, *r_m_* = 0.097 mm, *r_n_* = 45 mm, *h_n_* = 0.5 mm; (**m**) *v* = 0.050 mm/s, *W* = 2800 r/min, *r_m_* = 0.097 mm, *r_n_* = 45 mm, *h_n_* = 0.5 mm; (**n**) *v* = 0.050 mm/s, *W* = 3000 r/min, *r_m_* = 0.097 mm, *r_n_* = 45 mm, *h_n_* = 0.5 mm; (**o**) *v* = 0.050 mm/s, *W* = 3200 r/min, *r_m_* = 0.097 mm, *r_n_* = 45 mm, *h_n_* = 0.5 mm.

To better explore the relationship between the surface quality of the alumina ceramic slices and the normal sawing force, a NewView9000 model optical profiler was used to detect alumina ceramic slices with a diameter of 10 mm under normal temperature conditions, and the detection results are shown in Figure 13.

As shown in Figure 13, while other process parameters remain unchanged, as the spindle speed increased, the maximum normal sawing force gradually decreased, resulting in a gradual decrease in the surface roughness of the alumina ceramic chips. The maximum normal sawing force was positively correlated with the surface roughness of the chips. In future cutting experiments, the surface quality of chips can be further predicted by predicting the maximum normal sawing force during ultrasonic-assisted internal circular sawing of hard and brittle materials.

## 5. Conclusions

Based on a six-axis force sensor, an ultrasonic-assisted inner diameter saw cutting force model was proposed to improve surface quality. The correctness of the model in this paper was verified by comparing experimental data, and the influence of various process parameters on the normal sawing force was analyzed.

(i) A novel ultrasonic-assisted force model for inner diameter sawing was proposed by using the six-axis forces data sampled in processing;
(ii) Spherical abrasive particles were applied in the sawing force model to improve prediction accuracy. For the processing example with alumina ceramics, the mean value and variance of the proposed model’s prediction error were reduced by 5.08% and 2.56% compared to the regular tetrahedral abrasive model;
(iii) The highest normal sawing force peak could be obviously reduced and the surface quality of the slices significantly improved with the proposed sawing force model by adjusting the process parameters.

Due to the complexity of the engineering implementation of ultrasound-assisted inner diameter slice machining technology, the work in this paper can be improved, including improving the universality of the model for machining different materials, considering the influence of temperature and chip fluid under unconventional working conditions, and combining the limitations of the model assumptions in this paper.

## Figures and Tables

**Table 1:** Orthogonal experimental design scheme.

Case	Spindle Speed W r/min	Feed Rate *f_j_* mm/s	Radius of Spherical Abrasive Particles r_m_ mm	Amplitude A_f _ μm
1	2400	0.083	0.106	3.2
2	2400	0.05	0.097	3.8
3	2400	0.033	0.065	2.5
4	2400	0.033	0.085	4.4
5	2400	0.067	0.074	5.5
6	2600	0.05	0.065	4.4
7	2600	0.033	0.106	5.5
8	2600	0.083	0.074	3.8
9	2600	0.067	0.097	2.5
10	2600	0.033	0.085	3.2
11	2800	0.067	0.065	3.2
12	2800	0.033	0.106	3.8
13	2800	0.083	0.097	4.4
14	2800	0.05	0.085	5.5
15	2800	0.033	0.074	2.5
16	3000	0.033	0.074	4.4
17	3000	0.033	0.097	3.2
18	3000	0.083	0.065	5.5
19	3000	0.067	0.085	3.8
20	3000	0.05	0.106	2.5
21	3200	0.067	0.106	4.4
22	3200	0.05	0.074	3.2
23	3200	0.033	0.065	3.8
24	3200	0.083	0.085	2.5
25	3200	0.033	0.097	5.5

## Data Availability

Not applicable.
